# Supplementary material for: Metagenomic binning with assembly graph embeddings
Source: Bioinformatics. 2022 Aug 16;38(19):4481–7. doi: 10.1093/bioinformatics/btac557 (PMC9525014; doi:10.1093/bioinformatics/btac557)
Supplement: btac557_supplementary_data [file btac557_supplementary_data.zip › amber outputs.zip!/amber outputs/simHC/genome/purity_completeness_per_bin.pdf]

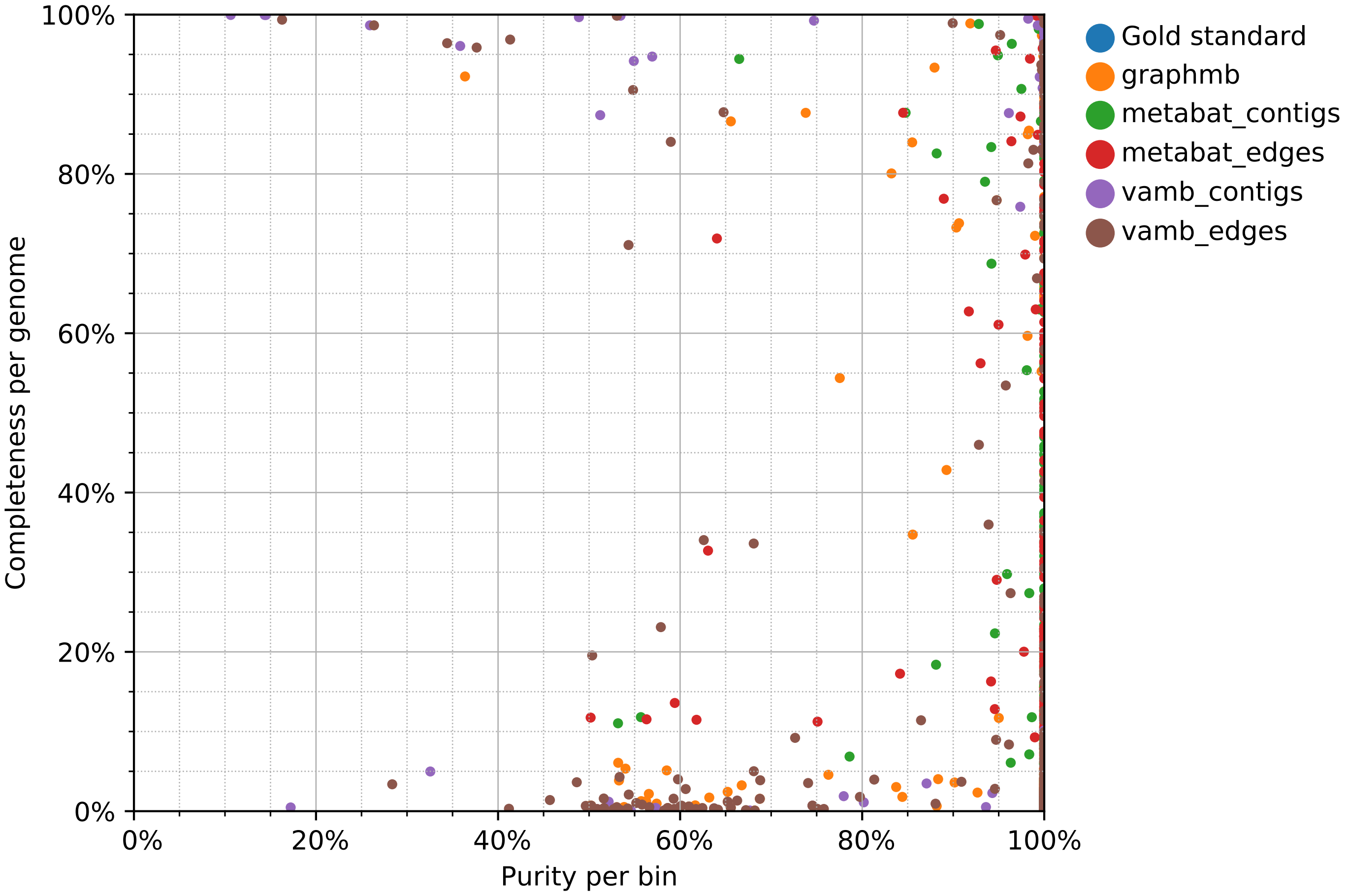

Completeness per genome
Purity per bin
0%
20%
40%
60%
80%
100%
Gold standard
graphmb
metabat_contigs
metabat_edges
vamb_contigs
vamb_edges
